# Observational Analyses of Ex Vivo Native American Platelet Responses

**DOI:** 10.3390/ijms252211990

**Published:** 2024-11-08

**Authors:** Krista Goerger, Madison Caldwell, Grace Biermann, Fatima Besh, Tanner Flickema, Pramit Patel, Karla Abbott, Michael Holinstat, Mark K. Larson

**Affiliations:** 1 Department of Pharmacology, University of Michigan, Ann Arbor, MI 48109, USA; kgoerger@umich.edu (K.G.); mbcald@med.umich.edu (M.C.); mholinst@med.umich.edu (M.H.); 2 Biology Department, Augustana University, Sioux Falls, SD 57197, USA; glsteinmeyer19@ole.augie.edu (G.B.); fabesh21@ole.augie.edu (F.B.); ttflickema20@ole.augie.edu (T.F.); ppatel19@ole.augie.edu (P.P.); 3 Nursing Department, Augustana University, Sioux Falls, SD 57197, USA; karla.abbott@augie.edu

**Keywords:** platelets, platelet activation, platelet agonists, thrombosis, Native American

## Abstract

Platelet activation plays an essential role in clot formation to prevent blood loss following vascular damage. In pathologic conditions, platelet activation can lead to obstructive clots, disrupting blood flow and resulting in thrombosis. Native Americans suffer disproportionately from arterial disease and previous research has shown that Blacks are enriched in genetic polymorphisms that correlate with higher platelet reactivity contributing to an increased risk for thrombosis. Therefore, the current study sought to determine phenotypic variations in Native American platelet responses following stimulation with agonists, simulating vascular damage. Several donors from a small cohort of Native Americans showed atypical robust platelet aggregation when stimulated with submaximal concentrations of agonists. Further, when comparing α-granule secretion, a specific marker of platelet activation, Native Americans were more likely to have elevated responses to multiple agonist conditions of stimulation compared to Whites. Interestingly, there were no noticeable differences in integrin activation between Native Americans and Whites. Our study is the first to observe elevated Native American platelet responses compared to Whites, supporting further mechanistic studies and investigation of treatment approaches for the prevention of thrombosis.

## 1. Introduction

Cardiovascular disease (CVD) still ranks as the leading cause of mortality and morbidity worldwide [1,2], with arterial thrombosis making up the majority of the total cases [3]. Among the multitude of contributing factors, blood platelet reactivity is a key cellular component in the development of ischemic arterial disease [4]. Following vascular injury, platelets adhere to exposed subendothelial matrix proteins, such as collagen, at the site of damage [5]. Platelet adhesion triggers the initial activation in which platelets rapidly undergo signaling cascades, leading to shape change, granule secretion, and thrombin generation to further amplify activation [6]. Activated platelets then cross-link via integrins, resulting in platelet aggregation and the formation of a hemostatic plug formation, preventing further blood loss [7]. However, under pathologic conditions, platelet activation occurs independent of external insult and is often more chronic in nature [8]. As such, platelet reactivity under these conditions can lead to sudden arterial thrombosis and cause disruptions to normal blood flow. The importance of platelet function in CVDs is illustrated by the prevalence of anti-platelet therapies (e.g., aspirin, clopidogrel, ticagrelor), which are given to an estimated five million patients in the USA alone [9,10,11]. The role of platelets in other disease conditions, such as disruptive micro-aggregation in response to SARS-CoV2 infection [12], also highlights the need to understand how platelets respond to microenvironmental disturbances in pathologic conditions.

Interestingly, there is substantial variation in platelet responsivity across human populations [13,14]. Further, prior studies have shown that Blacks express specific genetic polymorphisms in their platelet receptors, exhibiting elevated platelet activation levels in response to specific platelet agonists compared to Whites [15,16]. Importantly, this elevated response impacts the utility of certain anti-platelet medications [17]. The hyperreactive allele is enriched, but not limited to those of African descent. Allelic variation in liver enzymes can also alter the effectiveness of anti-platelet medications, as Asians and Pacific Islanders expressing a polymorphism in their liver enzyme gene experience a reduction in the efficacy of commonly used anti-platelet medications [18]. In all, these findings illustrate understanding platelet responsivity and genetic variations that influence platelet function and drug effects on platelets is necessary to make accurate treatment determinations for conditions where the risk of platelet hyperreactivity is present.

Another ethnic population with elevated levels of CVD is Native Americans [19]. While there are substantial differences across the various Native American populations in North America, as an ethnic group, they exhibit higher rates of disease burden, particularly with regard to arterial disease [20]. However, the degree to which we understand the pathophysiological risk factors in Native Americans such as platelet reactivity is lacking, significantly impairing the ability to provide adequate treatment in the face of other substantial health care disparities. One study characterized baseline platelet activation [21], but to our knowledge, there has not been any attempt made to characterize the sensitivity of Native American platelets in response to agonists simulating vascular damage. Here, we conducted a small observational pilot study with two cohorts of Native Americans to survey platelet responses. These novel studies are a means to identify potential phenotypic variations that can be used to guide future mechanistic studies and treatment approaches.

## 2. Results

### 2.1. Participant Characteristics

A total of forty-six Native American donors (predominantly self-identifying as Lakota Sioux) were recruited over two periods at Augustana University and the Urban Indian Health Clinic in Sioux Falls, South Dakota. A limited set of physical and social characteristics of the donors are described in Table 1, though the small sample sizes resulted in a lack of power to statistically analyze any correlation between the characteristics and the platelet responses. For the platelet aggregation cohort (n = 15), 27% were males and 73% were females, while the flow cytometry cohort (n = 31) donors were 53% males and 47% females. The median age of the donors was 32 and 37 years, respectively. For both cohorts, about 80% of donors received their high school diploma. In addition, more than half of the donors (57% and 74.2%) were unemployed or had a personal income of less than $20,000 at the time of the study. Approximately 50% of the donors across both cohorts have dependents and have health insurance. Additionally, in the flow cytometry cohort, 32% consumed a moderate level of alcohol (1–2 drinks/day) and 16% consumed alcohol at a more excessive rate (3–10 drinks/day). The majority of the donors do not consume alcohol daily, with 86% of the platelet aggregation cohort and 52% of the flow cytometry cohort consuming zero alcoholic drinks each day.

### 2.2. Differences in Platelet Aggregation

**Figure 1:**
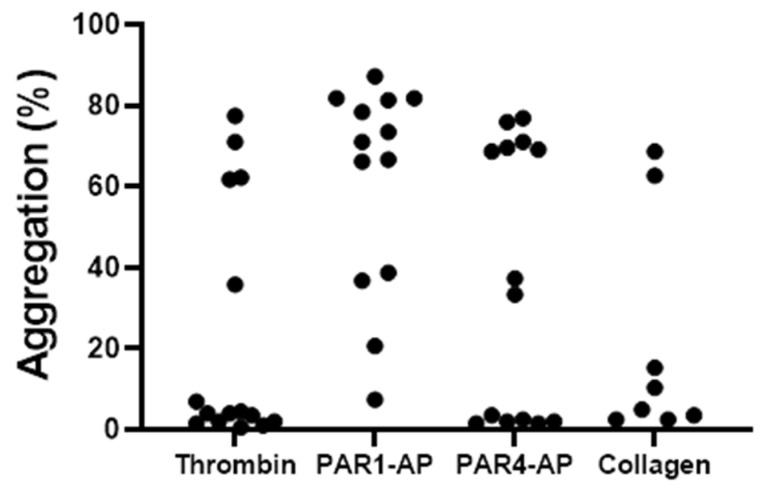
Differences in platelet aggregation within Native Americans: washed platelets from Native Americans (n = 8–15) were stimulated with 0.25 nM thrombin, 25 µM PAR4-AP, 1 µM PAR1-AP, or 0.375 µg/mL collagen. All concentrations are considered to be submaximal doses. Aggregation was measured using light transmission aggregometry for 10 min and reported as maximum percent aggregation.

To examine overall platelet function in Native Americans, agonist-induced platelet aggregation was assessed in response to several agonists. Washed platelets were prepared and stimulated with 0.25 nM thrombin, 25 µM PAR4-AP, 1 µM PAR1-AP, or 0.375 µg/mL collagen. The concentrations are submaximal based on the response rate in non-Native Americans in other studies under the same conditions [22,23,24]. Based on prior studies, we defined high responders to aggregation as those resulting in greater than 60% aggregation and low responders as those resulting in less than 40% aggregation following agonist stimulation [13]. For each agonist, a subset of Native Americans was observed in both the high- and low-aggregation-responding groups following stimulation with submaximal concentrations (Figure 1). The stimulation with PAR1-AP resulted in the highest number of participants with high responses (69%) compared to thrombin (26%), PAR4-AP (43%), and collagen (25%). These findings suggest that Native Americans exhibit a heightened propensity for robust platelet responses compared to White subjects, prompting us to further investigate this phenomenon using additional agonists at varying concentrations in flow cytometric assays.

### 2.3. Differences in Platelet α-Granule Secretion

**Figure 2:**
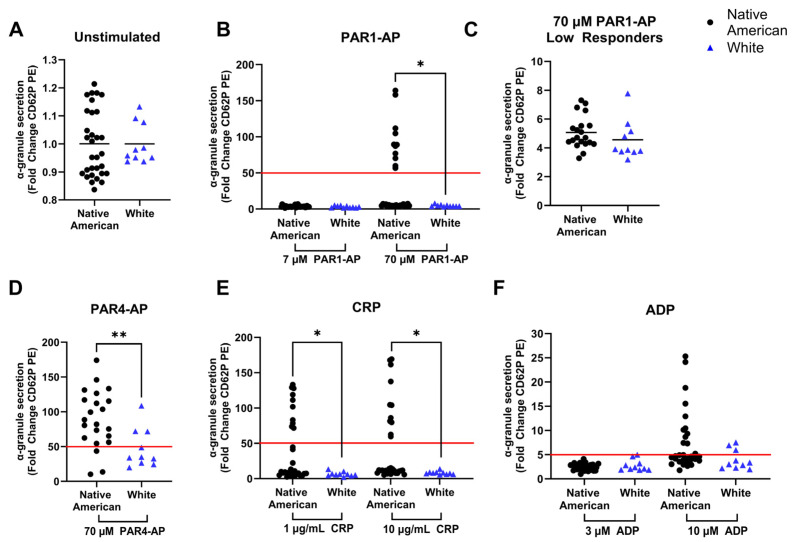
Increased platelet α-granule secretion in Native Americans relative to Whites: Stimulated platelet α-granule secretion is increased in subsets of Native Americans (n = 31) relative to Whites (n = 10). (**A**) Unstimulated α-granule secretion was measured to determine basal platelet activity. Agonist-induced α-granule secretion was assessed following stimulation with (**B**) PAR1-AP (7 and 70 µM), (**D**) PAR4-AP (25 µM), (**E**) CRP (1 and 10 µg/mL), and (**F**) ADP (3 and 10 µM) for 20 min. (**C**) Donors with low responses below the threshold (red line) were stimulated with 70 µM PAR1-AP. Data are presented as fold change relative to resting platelets to account for variations in unstimulated platelets. Black lines represent the mean. Red line represents the threshold to determine high responders. An unpaired two-tailed t-test was performed to determine significance (* *p* < 0.05, ** *p* < 0.01).

Since the aggregation results suggested variations in platelet responses within Native Americans, platelet reactivity was measured in a larger cohort of Native Americans with a side-by-side comparison to participants self-identifying as White. To examine specific outcomes of platelet activation more precisely, α-granule secretion was measured by flow cytometry in unstimulated and stimulated platelets in the presence of an antibody that selectively binds CD62P (P-selectin). In resting platelets, P-selectin is located intracellularly in storage vesicles called α-granules. Platelet activation results in the secretion of α-granules to reinforce platelet activation and clot formation, and P-selectin is subsequently found on the surface of the platelet, allowing for its detection [25]. Prior studies have demonstrated chronic inflammation increases basal levels of P-selectin expression on the surface of the platelet [26], but we found no difference in platelet α-granule secretion in resting platelets between the Native American and White cohorts (Figure 2A). All agonist conditions, consisting of low and high doses of PAR1-AP, PAR4-AP (one dose only), CRP, and ADP, increased α-granule secretion and platelet activation in all donors. As a whole the Native American cohort demonstrated elevated α-granule secretion following stimulation with 70 µM PAR1-AP, 70 µM PAR4-AP, and both 1 and 10 µg/mL CRP compared to the White cohort.

To further assess abnormal platelet reactivity within the groups, α-granule secretion responses were categorized as a high response if activation resulted in >50-fold change for the PAR1-AP, PAR4-AP, and collagen agonists and >5-fold change for the weaker agonist ADP. This separation of responses is demarcated by the red line present throughout Figure 2. A subset of Native Americans is observed as high responders in most conditions tested. The low responders exhibited a lower amplitude of P-selectin expression, yet the Native American and White donors in this category are indistinguishable from each other (Figure 2C). However, across multiple agonist conditions, a subset of predominantly Native American donors responded with more robust levels of CD62P expression, indicating a higher level of α-granule secretion (Figure 2B,D–F). In contrast, White donors had very few high responders.

**Figure 3:**
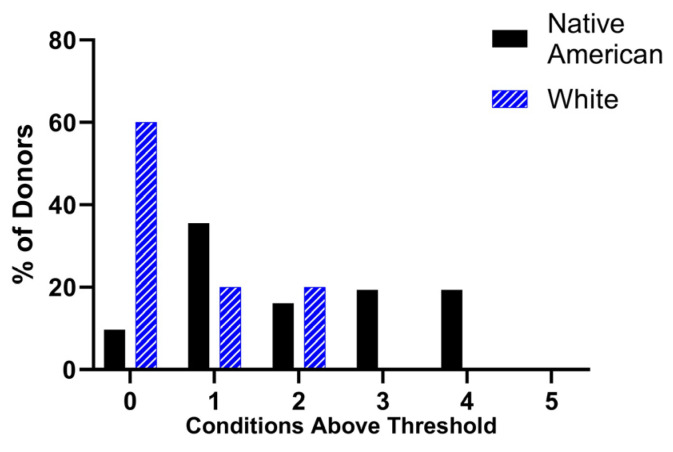
Frequency of high responses between Native Americans and Whites for α-granule secretion: Individual donors with α-granule secretion levels above the threshold were marked as high responders for each of the seven separate agonist conditions (Figure 2B,D–F). The total conditions of high responses for each subject were recorded and the frequency of responses in Native Americans (n = 31) and Whites (n = 10) are presented as percent of total donors.

To compare the responses between the groups, an assessment of which donors displayed a high response to α-granule secretion and how often each donor fell into the high response category in the conditions in Figure 2 (above the red line) was conducted. Each donor’s response to the seven separate agonist conditions was marked as high or low, and the number of conditions a donor exhibited high responses to was recorded. No donors showed high responses to five or more out of seven separate agonist conditions, but Native American donors had an increased frequency of high responses to three or four conditions. In contrast, 60% of White donors had low responses in all seven conditions (Figure 3).

### 2.4. Differences in Platelet Integrin α_IIb_β_3_ Activation

**Figure 4:**
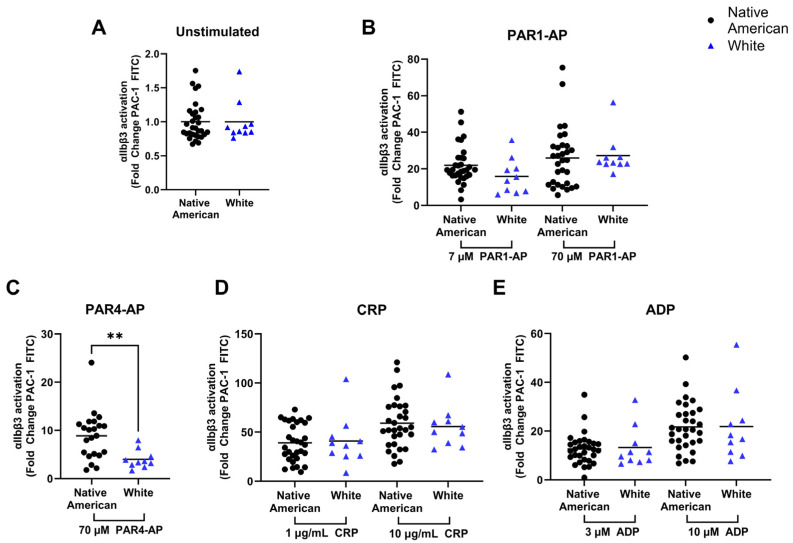
Variations in integrin α_IIb_β_3_ activation in platelets from Native Americans relative to Whites: Integrin α_IIb_β_3_ activation in Native American (n = 31) and White (n = 10) donors was measured 20 min after stimulation with various agonists: (**A**) unstimulated, (**B**) 7 and 70 µM PAR1-AP, (**C**) 70 µM PAR4-AP, (**D**) 1 and 10 µg/mL CRP, and (**E**) 3 and 10 µM ADP. Data are presented as fold change relative to resting platelets to account for variations in unstimulated platelets. Black lines represent the mean. An unpaired two-tailed t-test was performed to determine significance (** *p* < 0.01).

Using the same flow cytometric measurement as in Figure 2, Native American and White donors were also assessed for integrin α_IIb_β_3_ activation, another measure of platelet activation, in unstimulated and stimulated platelets (Figure 4). Integrin α_IIb_β_3_ is the most abundant platelet surface protein and plays a critical role in facilitating platelet aggregation. Upon platelet activation, integrin α_IIb_β_3_ undergoes a conformational change to an active form that allows platelet cross-linking [7]. The conformational change can be directly measured using the PAC-1 antibody that only binds the active conformation of the protein. The Native American and White groups showed similar levels of basal levels of integrin α_IIb_β_3_ activation (Figure 4A). Across most agonist conditions, there were no differences between Native Americans and Whites for integrin activation (Figure 4), except for platelets stimulated with PAR4-AP (Figure 4C).

## 3. Discussion

Since the Strong Heart Study was initiated in the 1980s, it is increasingly clear that Native Americans suffer disproportionately from CVDs [27]. However, in the time since the longitudinal study was launched, there has been little research investigating the prospect that platelet reactivity could be a contributing factor to the elevated risk. Here, we show, for the first time, evidence of hyperactive Native American platelet responses to agonists, simulating the platelet response to vascular injury. Like other ethnic populations with elevated levels of CVD, we see evidence in small cohorts of donors that Native Americans are more likely to have hyperreactive platelet responses. Our initial analysis of platelet aggregation showed that while some Native American donors had low responses to submaximal agonist concentrations, 25–69% of the donors had atypically high responses to stimulation (Figure 1). This is increased compared to a previous larger study including African American, White, Asian, and Hispanic participants in which a minority (7–26%) of donors exhibited atypical robust aggregation in response to submaximal concentrations of agonists [13]. While no definitive conclusion can be drawn due to the small sample size, these results suggest Native Americans may be more likely to have atypical robust platelet responses.

The measurements of the second cohort focused on more specific and quantifiable elements of platelet activation. When analyzing α-granule secretion following platelet activation, we observe an upward shift in platelet responsiveness to agonists in the Native American cohort compared to the White cohort tested (Figure 2). While many Native American responses were indistinguishable from White subjects, Native Americans were more likely to have elevated responses across multiple agonist conditions. Although we cannot rule out the presence of these elevated responders as an artifact of the small sample size, this finding is consistent with previous research suggesting that platelet hyperreactivity is a global characteristic, meaning an individual with a hyperreactive response to one agonist tends to demonstrate a similar response to other agonists [13]. The elevated presence of CD62P on activated platelets strongly suggests that subsets of Native Americans may be more susceptible to rapid cross-cellular interactions that occur when platelets are activated. In a pathological state such as atherosclerosis, this could readily accelerate and exacerbate the disease.

Intriguingly, the elevated responses were not observed in all aspects of platelet activation. Unlike the measure of overall aggregation measured in Figure 1, we generally see that integrin α_IIb_β_3_ activation is similar between Native Americans and White donors. The exception to this was seen in response to the PAR4-thrombin receptor activating peptide (Figure 4C). This is notable, as this receptor has been characterized in Blacks as being more responsive to agonist stimulation [28], suggesting that similar variations of this receptor might also be enriched in Native American populations. However, the general lack of correlation between the overall platelet aggregation data to the integrin α_IIb_β_3_ activation data suggests that other factors besides integrin α_IIb_β_3_ that control aggregation are worth considering in more detail. Overall, the results suggest that platelet differences present in subsets of Native Americans may be limited to certain aspects of platelet function, rather than across all elements of platelet activity. Further, variations in Native American platelets are potentially more diverse than the mechanistic measures contained in our study, and additional follow-up studies are required to determine other contributing factors that lead to increased platelet reactivity.

A major limitation of the current study is its small sample size. While we report some demographic characteristics of the Native American cohorts in Table 1 to provide background information on the donors, the sample size is too small to interpret any meaningful statistical correlation of age, gender, or environmental factors that may be contributing to platelet reactivity. However, we feel it is noteworthy that the subsets of “high responders” in different agonist conditions in Figure 2 are not the same individuals each time. In fact, only ~10% of the Native American donors were never a high responder in the seven conditions, while 60% of the White donors were never high responders (Figure 3). The range of demographics, along with the distribution of high platelet reactivity across the majority of the Native American donors, implies that socioeconomic demographic factors cannot entirely account for the observed elevated responses. Since genetic variation is known to also contribute to reactivity in other populations, follow-up studies are needed to further characterize the responses seen here. Further, platelet surface receptor expression was not quantified in this study and elevated receptor expression could contribute to platelet hyperreactivity; therefore, this needs to be included in future studies.

Further studies are also needed to be able to apply what is learned from these studies of platelet reactivity as a means to inform patient care [29]. Native Americans already suffer from disparities in health care access, treatment, and outcomes, and any efforts to combat those disparities need to be informed to ensure they have the capacity to be successful. In many respects, this approach is consistent with the idea of personalized medicine, and platelet reactivity may be a tool that can be used to better treat the individual as we aim to improve the health of whole populations [30]. Regardless of the extrinsic or intrinsic factors resulting in the elevated responses, the evidence from other populations with higher responses has substantial implications for how this elevation might influence treatment options. Native Americans are already faced with substantial disparities when it comes to health care outcomes, and the prevention of elevated platelet responses may also need to be considered in any approach that aims to improve those outcomes.

## 4. Materials and Methods

### 4.1. Human Subjects

All human subject procedures and protocols were approved by the Augustana University IRB (Study FA17.04) and with the approval of the South Dakota Urban Indian Health Clinic in Sioux Falls, South Dakota. All subjects were over 18 years of age, and written informed consent was obtained from subjects prior to enrollment in the study. Subjects self-identified as healthy and reported their races as Native American or Non-Hispanic White. Participants declared they had not taken any platelet-modifying medication in the previous 48 h (or two weeks in the case of aspirin). A total of 46 Native American donors were enrolled, with 15 Native Americans enrolled for the platelet aggregation cohort and 31 Native American and 10 White donors enrolled in the flow cytometry cohort.

### 4.2. Blood Isolation and Analysis

Blood was drawn via venipuncture into 5 mL vacutainers with 3.2% sodium citrate as an anticoagulant and warmed to 37 °C for at least 30 min. Platelet activation was then measured largely as described by Huskens et al. [31]. Briefly, whole blood was diluted with HEPES-buffered saline solution (10 mM HEPES, 150 mM NaCl, 1mM MgSO_4_, and 5 mM KCl with pH of 7.4) in a 1:3 ratio and incubated for 10 min at 37 °C. For flow cytometry analyses, diluted blood was added to prepared reaction tubes containing a titrated agonist concentration to elicit an approximate EC_30_ (low dose) or EC_80_ (high dose). Agonists were adenosine diphosphate (ADP, Sigma, St. Louis, MO, USA), collagen receptor peptide (CRP, courtesy of Dr. Joe Aslan, Oregon Health Science University), thrombin receptor 1 activating peptide (PAR1-AP, Abcam, Waltham, MA, USA), and thrombin receptor 4 activating peptide (PAR4-AP, Abcam). Reaction tubes also contained both FITC-labeled PAC-1 antibody and PE-labeled anti-CD62P antibody (BD Biosciences, Franklin Lakes, NJ, USA). Diluted whole blood was incubated in reaction tubes for 20 min at 37 °C and then fixed in a 10:1 volume of 137 mM NaCl, 2.7 mM KCl, 1.12 mM NaH_2_PO_4_, 1.15 mM KH_2_PO_4_, 10.2 mM Na_2_HPO_4_, 4 mM EDTA, and 0.5% formaldehyde. Samples were then analyzed on an Accuri C6 flow cytometer (BD Biosciences) within one day of fixation. Data are presented as fold change relative to resting platelet fluorescent values to account for variation in unstimulated baseline readings. Alternatively, washed platelets were prepared from the collected whole blood samples, and platelet aggregation was subsequently analyzed using a Chronolog Model 700 aggregometer as previously described [16]. Platelets were stimulated with collagen (Chronolog, Havertown, PA, USA) instead of CRP, thrombin (Chronolog), PAR1-AP, and PAR4-AP.

### 4.3. Statistical Analysis

Unpaired two-tailed *t*-tests were performed using the GraphPad Prism Software 10.3 (GraphPad Software, Boston, MA, USA) software for analysis.

## Figures and Tables

**Table 1:** Participant physical and social characteristics: donor characteristics are shown as number (percentage) for gender, education, personal income, dependents, health insurance, and alcohol consumption.

Characteristic	Platelet Aggregation (n = 15) ^1^	Platelet Flow Cytometry (n = 31) ^1^
Gender		
Male	4 (27%)	16 (53%)
Female	11 (73%)	14 (47%)
Age		
Median	32	37
Range	21–61	20–70
Education		
No High School Diploma	3 (21%)	6 (19%)
High School Diploma	11 (79%)	25 (81%)
Personal Income		
<$20,000/Unemployed	8 (57%)	23 (74%)
$20,000–$34,999	4 (29%)	0
>$35,000	2 (14%)	8 (26%)
Dependents		
Yes	8 (57%)	16 (52%)
No	6 (43%)	15 (48%)
Health Insurance		
Yes	7 (50%)	15 (50%)
No	7 (50%)	15 (50%)
Average Alcoholic Drinks Consumed per Day		
0	12 (86%)	16 (52%)
1–2	1 (7%)	10 (32%)
3–10	1 (7%)	5 (16%)

^1^ Size in categories vary from total donor numbers due to missing values.

## Data Availability

Data is contained within the article.

## References

[1] Tsao C.W., Aday A.W., Almarzooq Z.I., Anderson C.A.M., Arora P., Avery C.L., Baker-Smith C.M., Beaton A.Z., Boehme A.K., Buxton A.E. (2023). Heart Disease and Stroke Statistics-2023 Update: A Report From the American Heart Association. Circulation.

[2] Vaduganathan M., Mensah G.A., Turco J.V., Fuster V., Roth G.A. (2022). The Global Burden of Cardiovascular Diseases and Risk: A Compass for Future Health. J. Am. Coll. Cardiol..

[3] Roger V.L., Go A.S., Lloyd-Jones D.M., Adams R.J., Berry J.D., Brown T.M., Carnethon M.R., Dai S., de Simone G., Ford E.S. (2011). Heart disease and stroke statistics—2011 update: A report from the American Heart Association. Circulation.

[4] Koupenova M., Kehrel B.E., Corkrey H.A., Freedman J.E. (2017). Thrombosis and platelets: An update. Eur. Heart J..

[5] Jackson S.P. (2007). The growing complexity of platelet aggregation. Blood.

[6] Ruggeri Z.M. (1997). Mechanisms initiating platelet thrombus formation. Thromb. Haemost..

[7] Ni H., Freedman J. (2003). Platelets in hemostasis and thrombosis: Role of integrins and their ligands. Transfus. Apher. Sci..

[8] Ruggeri Z. (2002). Platelets in atherothrombosis. Nat. Med..

[9] The Top 200 Drugs of 2022. https://clincalc.com/DrugStats/.

[10] Thachil J. (2016). Antiplatelet therapy—A summary for general physicians. Clin. Med..

[11] Stanger L., Yamaguchi A., Holinstat M. (2023). Antiplatelet strategies: Past, present, and future. J. Thromb. Haemost..

[12] Nishikawa M., Kanno H., Zhou Y., Xiao T.H., Suzuki T., Ibayashi Y., Harmon J., Takizawa S., Hiramatsu K., Nitta N. (2021). Massive image-based single-cell profiling reveals high levels of circulating platelet aggregates in patients with COVID-19. Nat. Commun..

[13] Yee D.L., Sun C.W., Bergeron A.L., Dong J.F., Bray P.F. (2005). Aggregometry detects platelet hyperreactivity in healthy individuals. Blood.

[14] Lindkvist M., Fernberg U., Ljungberg L.U., Fälker K., Fernström M., Hurtig-Wennlöf A., Grenegård M. (2019). Individual variations in platelet reactivity towards ADP, epinephrine, collagen and nitric oxide, and the association to arterial function in young, healthy adults. Thromb. Res..

[15] Edelstein L.C., Simon L.M., Montoya R.T., Holinstat M., Chen E.S., Bergeron A., Kong X., Nagalla S., Mohandas N., Cohen D.E. (2013). Racial differences in human platelet PAR4 reactivity reflect expression of PCTP and miR-376c. Nat. Med..

[16] Tourdot B.E., Conaway S., Niisuke K., Edelstein L.C., Bray P.F., Holinstat M. (2014). Mechanism of race-dependent platelet activation through the protease-activated receptor-4 and Gq signaling axis. Arterioscler. Thromb. Vasc. Biol..

[17] Tourdot B.E., Stoveken H., Trumbo D., Yeung J., Kanthi Y., Edelstein L.C., Bray P.F., Tall G.G., Holinstat M. (2018). Genetic Variant in Human PAR (Protease-Activated Receptor) 4 Enhances Thrombus Formation Resulting in Resistance to Antiplatelet Therapeutics. Arterioscler. Thromb. Vasc. Biol..

[18] Beitelshees A.L., Thomas C.D., Empey P.E., Stouffer G.A., Angiolillo D.J., Franchi F., Tuteja S., Limdi N.A., Lee J.C., Duarte J.D. (2022). CYP2C19 Genotype-Guided Antiplatelet Therapy After Percutaneous Coronary Intervention in Diverse Clinical Settings. J. Am. Heart Assoc..

[19] Howard B.V., Lee E.T., Cowan L.D., Fabsitz R.R., Howard W.J., Oopik A.J., Robbins D.C., Savage P.J., Yeh J.L., Walty T.K. (1995). Coronary Heart Diseease Prevalence and Its Relation to Risk Factors in American Indians: The Strong Heart Study. The Strong Heart Study. Am. J. Epidemiol..

[20] Fabsitz R.R., Sidawy A.N., Go O., Lee E.T., Welty T.K., Devereux R.B., Howard B.V. (1999). Prevanence of peripheral arterial disease and associated risk factors in American Indians: The Strong Heart Study. Am. J. Epidemiol..

[21] Au N.T., Reyes M., Boyer B.B., Hopkins S.E., Black J., O’Brien D., Fohner A.E., Yracheta J., Thornton T., Austin M.A. (2017). Dietary and genetic influences on hemostasis in a Yup’ik Alaska Native population. PLoS ONE.

[22] Yamaguchi A., Stanger L., Freedman C.J., Standley M., Hoang T., Adili R., Tsai W.C., van Hoorebeke C., Holman T.R., Holinstat M. (2021). DHA 12-LOX-derived oxylipins regulate platelet activation and thrombus formation through a PKA-dependent signaling pathway. J. Thromb. Haemost..

[23] Yeung J., Adili R., Stringham E.N., Luo R., Vizurraga A., Rosselli-Murai L.K., Stoveken H.M., Yu M., Piao X., Holinstat M. (2020). GPR56/ADGRG1 is a platelet collagen-responsive GPCR and hemostatic sensor of shear force. Proc. Natl. Acad. Sci. USA.

[24] Yeung J., Apopa P.L., Vesci J., Stolla M., Rai G., Simeonov A., Jadhav A., Fernandez-Perez P., Maloney D.J., Boutaud O. (2013). 12-lipoxygenase activity plays an important role in PAR4 and GPVI-mediated platelet reactivity. Thromb. Haemost..

[25] Yang H., Lang S., Zhai Z., Li L., Kahr W.H., Chen P., Brkić J., Spring C.M., Flick M.J., Degen J.L. (2009). Fibrinogen is required for maintenance of platelet intracellular and cell-surface P-selectin expression. Blood.

[26] Koupenova M., Clancy L., Corkrey H.A., Freedman J.E. (2018). Circulating Platelets as Mediators of Immunity, Inflammation, and Thrombosis. Circ. Res..

[27] Howard B.V., Lee E.T., Cowan L.D., Devereux R.B., Galloway J.M., Go O.T., Howard W.J., Rhoades E.R., Robbins D.C., Sievers M.L. (1999). Rising Tide of cardiovascular disease in American Indians. The Strong Heart Study. Circulation.

[28] Edelstein L.C., Simon L.M., Lindsay C.R., Kong X., Teruel-Montoya R., Tourdot B.E., Chen E.S., Ma L., Coughlin S., Nieman M. (2014). Common variants in the human platelet PAR4 thrombin receptor alter platelet function and differ by race. Blood.

[29] Lordkipanidzé M., So D., Tanguay J.F. (2016). Platelet function testing as a biomarker for efficacy of antiplatelet drugs. Biomark. Med..

[30] Yeung J., Li W., Holinstat M. (2018). Platelet Signaling and Disease: Targeted Therapy for Thrombosis and Other Related Diseases. Pharmacol. Rev..

[31] Huskens D., Sang Y., Konings J., van der Vorm L., de Laat B., Kelchtermans H., Roest M. (2018). Standardization and reference ranges for whole blood platelet function measurements using a flow cytometric platelet activation test. PLoS ONE.

